# The use of biodegradable temporising matrix (BTM) for facial unit reconstruction with adjuvant radiotherapy—A case study

**DOI:** 10.1016/j.jpra.2024.04.001

**Published:** 2024-04-06

**Authors:** T.A. Buick, A.M. Pathak, D.J. Jordan

**Affiliations:** Ninewells Hospital and Medical School, Dundee, Scotland, United Kingdom

**Keywords:** BTM, Novosorb, Wound management, Skin substitute, BCC, Radiotherapy

## Abstract

Synthetic Biodegradable Temporising Matrix (BTM, NovoSorb; PolyNovo Biomaterials Pty Ltd, Port Melbourne, Victoria, Australia) has proven useful in the resurfacing of large burns,^1^ necrotising infection debridement^2^ and tumour excision with exposed bone.^3^ We present a case report of a large BCC invading three aesthetic subunits of the face which was successfully reconstructed with BTM, split-thickness skin graft with subsequent adjuvant radiotherapy due to the high risk nature of the BCC. We present our series of images illustrating the timeline of BTM, and the ability to achieve a good skin colour match with minimal contour deformity, even in the event of post operative radiotherapy use.

## Introduction

Basal cell carcinoma (BCC) is the most common form of skin cancer, primarily affecting the head and neck regions. The gold standard treatment is surgical excision while preserving functional and aesthetic outcomes. These aims are more complex with BCC's affecting the face where the optimal aesthetic result can be more challenging. Likewise, subsequent radiotherapy is known to further injure skin graft reconstructions, particularly split-thickness skin grafts. As a result, flap reconstruction is often preferred where radiotherapy is considered post-operatively.

A promising option for achieving satisfactory aesthetic outcomes is reconstruction using synthetic matrices. Biodegradable Temporising Matrix (BTM), developed in Australia in 2004,^2^ has shown promise as a novel dermal substitute used in complex burns healing, deep infected wounds, reconstruction of subfascial defects and necrotising fasciitis.^1^^,^^2^^,^^4^ BTM has been available in the UK since 2019.^5^

It is a 2-mm thick synthetic dermal template, with a biodegradable polyurethane open-cell foam bonded via a polyurethane adhesive layer to a superficial transparent nonbiodegradable polyurethane sealing membrane,^6^^,^^7^ requiring a two stage wound reconstruction in the mainstay. In the first stage, BTM provides physiological wound cover, with fenestration to avoid accumulation of underlying materials, into which cells and blood vessels migrate to form a neodermis. As granulation tissue grows into the foam matrix, the foam itself biodegrades, ultimately leaving a graftable surface. A split thickness skin graft is often considered for the second stage.^8^ It has antibacterial properties and does not supply nutrients from animal tissue in comparison to other dermal substitutes and been demonstrated to be cost effective with greater vascularisation and inflammatory response when compared with other dermal substitutes.^9^

## Case report

**Figure 1:**
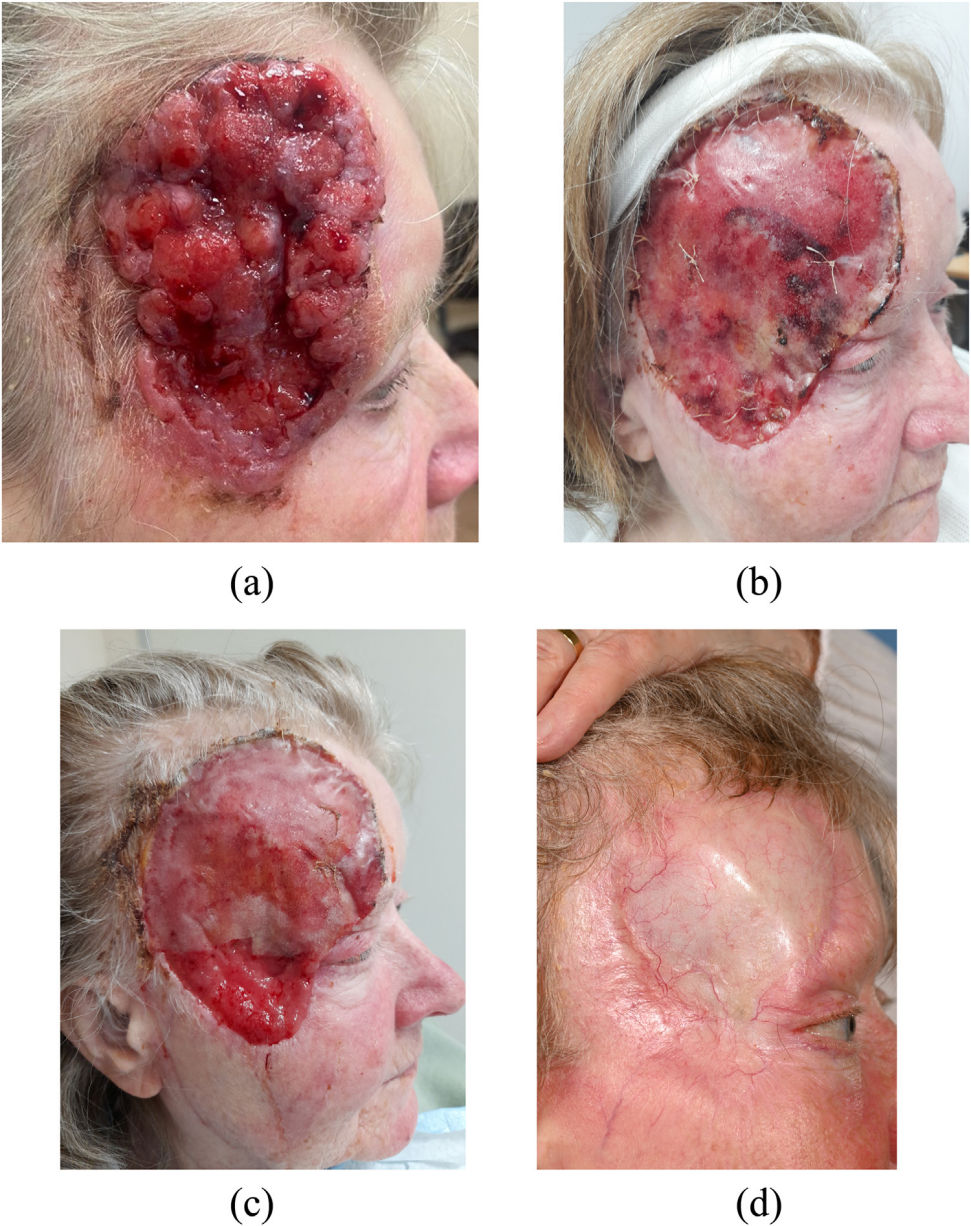
A visual timeline of BTM healing showing – (A) the pre-operative BCC, (B) appearance at day 20, (C) at 1 month and (D) 10 months following skin graft and 3 months following radiotherapy.

A 70 year old lady presented with a lesion on the right temple towards the end of the COVID-19 isolation period in the UK. The lesion had been slowly growing for a number of years and she had developed a comb over fringe to successfully hide it from family and friends. The lesion had grown anteriorly to involve the lateral canthus of the right eye, inferiorly to the zygomatic ridge, posteriorly into the hair line and superiorly to the frontal scalp hair line (Figure 1A). Biopsy confirmed this to be a nodular and infiltrative type BCC. She underwent pre-operative CT staging to rule out bone involvement.

The patient declined free or local flap reconstructions and surgery was expected to involve excision of the periosteum due to the fixed nature of the lesion and the CT findings. The patient was offered a two-stage reconstruction where the BCC was excised and dressed with BTM initially. The excision was completed down to bone at the orbit and over the zygoma with periosteum excised for over 70 % of the defect. The BTM dressing was placed over the whole area and secured with vicryl rapide^(TM)^ peripherally and quilting stitches centrally. A foam tie-over dressing was utilised for 5 days where after, the patient returned for weekly dressing changes.

Once the silicone top layer of BTM had started to delaminate a second stage split-thickness skin graft operation was planned. In theatre the silicone layer of BTM was removed and the underlying granulating sponge was cleaned gently with a saline soaked swab. A split thickness skin graft harvested from the lateral thigh was secured with a foam tie-over dressing. The graft was reviewed as standard at 1 week. The BCC was adequately excised but due to several high risk features, the area was subsequently treated with postoperative radiotherapy.

## Discussion

The wound healing potential of BTM has been well described in the literature, but to our knowledge this is the first use in resurfacing a significant facial skin cancer with subsequent radiotherapy in the UK. The technicalities of placing the BTM intra-operatively are no more difficult than other dermal substitutes. However, we found the nursing support and experience post operatively was critical to achieve this result.

At day 20 some cellulitis/superficial infection of the wound edge and BTM was evident (Figure 1B). This was managed with oral antibiotics and milking of the wound bed through small incisions of the silicone. With alternative dermal substitutes (or even a one stage reconstruction) this appearance could signal an infected site and risk failure of the matrix. In contrast, this was easily managed when using BTM by simply irrigating the area with saline and leaving it in place, meaning the ongoing granulation could continue without significant morbidity. This granulation layer is illustrated in Figure 1C where the BTM foam has degraded and left a well vascularised bed for grafting. At the second stage, this layer was cleaned lightly taking care to not disturb the lightly adherent tissue. Skin graft take was excellent and the build up of the granulation bed resulted in minimal contour defect (Figure 1D). In addition, this limited the expected desquamating effect of post operative radiotherapy. There was some breakdown around 6 weeks following radiotherapy but at 6 months the area had fully healed (Figure 1D).

It is possible this defect could have been covered by a simple skin graft, however as a significant portion of the wound bed was denuded bone we wanted to ensure a graftable bed. By allowing granulation tissue to build up with the aid of BTM, a robust skin graft then survived post operative radiotherapy.

We still have limited and early use of BTM in our unit and so dressing changes were supervised by the operating consultant. For subsequent patients we have found the training of the dressing nursing staff, as with other plastic surgery cases, key to success but no different to standard post-operative graft checks. Removal of the silicone layer 1D does lead to some disorganised granulation formation however.

## Summary

To our knowledge this is the first large aesthetic subunit that has been reconstructed with BTM for skin cancer excision and subsequently treated with radiotherapy. The aesthetic result was excellent and the patient was spared the donor morbidity of a free flap. We found that one key stage in dressing management was tolerating a certain level of infection under the top silicone layer. This was managed by perforating the top layer and irrigating with saline. This allowed the wound bed to develop a thick layer of granulation tissue and ultimately led to a wound bed with no contour deformity on the face, requiring only a split-thickness (rather than the usually favoured full-thickness skin graft) on the face.

## Statements

Patient consent was sought for this specific publication and publication of images.

## Ethical approval

Not required.

## Declaration of competing interest

None declared.
